# Circulating Immune-Cell Profiles and Diffuse Idiopathic Skeletal Hyperostosis: A Community-Based Cross-Sectional Study

**DOI:** 10.3390/jcm15218458

**Published:** 2026-10-30

**Authors:** Yu Li, Gentaro Kumagai, Sunao Tanaka, Kairo Wada, Takaaki Nakano, Sho Kaneko, Kazushige Koyama, Yasuyuki Ishibashi, Koichi Murashita, Kanichiro Wada

**Affiliations:** 1 Department of Orthopaedic Surgery, Hirosaki University Graduate School of Medicine, 5 Zaifu-cho, Hirosaki 036-8562, Aomori, Japan; 2 The Center of Well-being Regional Society Innovation, Hirosaki University Research Institute of Health Innovation, 5 Zaifu-cho, Hirosaki 036-8562, Aomori, Japan

**Keywords:** diffuse idiopathic skeletal hyperostosis, natural killer cells, innate immunity, flow cytometry, cardiometabolic abnormalities

## Abstract

**Objectives:** Diffuse idiopathic skeletal hyperostosis (DISH) is associated with cardiometabolic abnormalities, but its circulating immune-cell profile remains poorly characterized. This exploratory study examined immune-cell subsets and soluble inflammatory markers in relation to prevalent DISH. **Method:** Existing data from 526 participants in the 2021 Iwaki Health Promotion Project were analyzed using Firth-penalized logistic regression adjusted for age, sex, and visceral fat area. False-discovery-rate correction was applied jointly to eight immune-cell proportions and five inflammatory markers. Subsequent analyses characterized the CD16+CD56+ natural killer (NK)-cell association. **Results:** Forty-one participants had DISH. CD16+CD56+ NK-cell proportion was positively associated with DISH (OR per 10-percentage-point increase, 2.60; 95% CI, 1.81–3.73), whereas CD16−CD56− proportion was inversely associated (OR, 0.41; 95% CI, 0.29–0.58). Both associations remained significant after multiple-testing correction. Associations involving CD4+CD8− helper T cells and IL-6 were nominally significant but did not survive correction. Additional adjustment for smoking and alcohol use yielded a similar NK-cell estimate. Both NK-cell proportion and count were associated with DISH separately, but only the proportion remained significant when modeled together. Proportions differed across radiographic categories but were not associated with ossification burden among participants with DISH. **Conclusions:** DISH was associated with differences in circulating immune-cell proportions. These exploratory findings require independent confirmation and do not establish disease specificity, clinical biomarker utility, or a causal role in ossification.

## 1. Introduction

Diffuse idiopathic skeletal hyperostosis (DISH) is characterized by flowing anterolateral spinal ossification and bridging of adjacent vertebral segments [1,2]. Although its structural complications, including spinal ankylosis and fracture susceptibility, are well recognized [3], the systemic processes associated with DISH remain incompletely understood. Associations with metabolic syndrome, central adiposity, elevated blood pressure, and cardiovascular morbidity suggest a broader cardiometabolic phenotype [4,5,6]. An association with insulin resistance has also been reported, although it attenuated after adjustment for metabolic syndrome components [7]. These observations raise the question of whether DISH is accompanied by systemic alterations beyond conventional metabolic abnormalities.

Metabolic dysfunction and immune regulation are closely interconnected. Experimental studies have implicated adaptive and innate immune cells in obesity-related inflammation and impaired insulin sensitivity [8,9]. In DISH, reports of low-grade inflammation and altered circulating Dickkopf-1 and adiponectin concentrations have likewise suggested links between systemic metabolic disturbances and bone-regulatory pathways [10,11,12,13]. Immune cells can also influence bone remodeling directly. In experimental arthritis, NK cells promoted osteoclast formation through interactions involving RANKL and macrophage colony-stimulating factor [14], whereas IL-15-activated NK cells inhibited bone erosion by killing mature osteoclasts in vitro [15]. These contrasting findings indicate that NK-cell effects on bone depend on cellular context and activation state. They provide a rationale for investigating immune-cell profiles in DISH, but do not establish a mechanism for the entheseal and ligamentous ossification characteristic of this disease.

Circulating lymphocyte subsets may provide information complementary to soluble inflammatory markers, but their associations with DISH have not been well characterized. Relative proportions and absolute counts describe different aspects of the circulating cellular profile: an increased proportion may reflect a higher count of the measured subset, lower counts of other populations, or both [16,17]. Examining both measures may therefore help distinguish statistical associations with relative cellular representation from associations with absolute abundance. Whether any observed differences are specific to DISH or reflect shared cardiometabolic exposures remains an open question.

This exploratory cross-sectional study examined associations of available circulating immune-cell subsets and soluble inflammatory markers with prevalent DISH in a community-based population. The aim was to characterize the circulating immune profile associated with DISH rather than establish a diagnostic biomarker or a causal cellular mechanism. Follow-up analyses characterized the observed CD16+CD56+ NK-cell association using relative and absolute measures, alternative covariate adjustments, and radiographic categories and ossification burden.

## 2. Methods

### 2.1. Study Design and Participants

This cross-sectional study used existing data from the June 2021 examination of the Iwaki Health Promotion Project, a community-based health examination program in Hirosaki, Japan [18]. A total of 536 volunteers participated from approximately 12,000 eligible residents of the Iwaki area. Recruitment targeted adults older than 20 years through telephone calls from public health nurses and advertisements in the mass media. With approval from the project, existing radiographic, clinical, anthropometric, biochemical, and immune-cell data were obtained from its database. No additional examinations, sample collection, or laboratory measurements were performed for this study. Eligibility for the analysis required interpretable whole-spine radiographs and available measurements for the exposures and principal adjustment variables.

Participants with ankylosing spondylitis or relevant postoperative spinal conditions were excluded because these conditions could interfere with radiographic classification. VFA entries recorded as “Not Applicable” were considered invalid and excluded from analyses requiring VFA; this value was not treated as a physiological measurement. Otherwise, participants were not excluded solely because they had extreme but valid immune-cell or metabolic measurements.

The present database-based study was approved by the Ethics Committee of Hirosaki University Graduate School of Medicine (approval no. 2022-007H; 3 April 2026) and was conducted in accordance with the Declaration of Helsinki. Access to and use of the study data were authorized by the Iwaki Health Promotion Project. Written informed consent had been obtained from participants before participation in the project.

### 2.2. Radiographic Assessment

Lateral whole-spine radiographs were independently evaluated by two orthopedic physicians blinded to metabolic and immune-cell measurements. Disagreements were resolved by consultation with a third physician and, when necessary, consensus discussion. Inter-reader reliability was assessed using Cohen’s κ for DISH classification, and a two-way random-effects, absolute-agreement, single-measure intraclass correlation coefficient for the ossification burden score (OBS); positive and negative agreement were additionally summarized for DISH classification, and Bland–Altman analysis assessed agreement in OBS.

DISH was classified using the radiographic criteria of Resnick and Niwayama [2]. EDISH was classified according to Kuperus et al. [19]. Participants with EDISH were included in the non-definite-DISH comparator group for the binary analysis and were evaluated separately in analyses of radiographic categories. Anterior spinal ossification at each available intervertebral level was graded from 0 to 3 (0, no definite ossification; 1, nonbridging new bone formation; 2, near-complete bridging; and 3, complete bridging) [19,20]. Segmental scores were summed to obtain the OBS. Analyses of OBS were restricted to participants with definite DISH and were considered exploratory.

### 2.3. Clinical and Metabolic Measurements

Clinical and metabolic variables included body mass index (BMI), waist circumference, visceral fat area (VFA), systolic and diastolic blood pressure (SBP and DBP), fasting glucose, fasting insulin, triglycerides (TG), and high-density lipoprotein cholesterol (HDL-C). VFA (cm^2^) was estimated using segmental multifrequency bioelectrical impedance analysis with an InBody 770 body composition analyzer. HOMA-IR was calculated as fasting insulin (µU/mL) × fasting glucose (mmol/L)/22.5, and the triglyceride–glucose (TyG) index as ln[fasting TG (mg/dL) × fasting glucose (mg/dL)/2]. Information on diabetes, dyslipidemia, hypertension, glucose-lowering therapy, lipid-lowering therapy, antihypertensive treatment, smoking history, and alcohol use was obtained from the project’s clinical and questionnaire records. Smoking history was classified as ever smoking, comprising current and former smoking, versus never smoking. Alcohol use was classified as current drinking versus no current drinking. Metabolic variables were grouped into glycemic, lipid, adiposity, hemodynamic, diagnosed-condition, and medication domains for sensitivity analyses.

### 2.4. Inflammatory Markers and Immune-Cell Variables

Laboratory measurements were obtained from the Iwaki project database and were performed within the project’s standardized examination procedures. The overall project framework has been described previously [18]. Available inflammatory markers comprised C-reactive protein (CRP), high-sensitivity CRP (hs-CRP), interleukin-6 (IL-6), tumor necrosis factor-α (TNF-α), and complement component C3. Values reported below a numerical reporting limit were assigned half that limit before analysis. CRP, hs-CRP, IL-6, and TNF-α were natural-log transformed because of their right-skewed distributions; C3 was analyzed on its original scale. Immune-cell data were supplied as recorded percentages and absolute counts. The available variables included the database-annotated CD16+CD56+ NK-cell population, the other CD16/CD56-defined components, CD4+CD8+ double-positive T cells, CD4−CD8+ cytotoxic T cells, CD4+CD8− helper T cells, and CD3−CD19+ B cells. The CD16−CD56− component was retained as a marker-defined variable without assigning a specific cellular lineage. Raw cytometry files and detailed 2021 laboratory metadata, including sample-processing information, antibody panels, instrumentation, gating hierarchy, percentage denominators, and assay-specific laboratory details, were not available for this secondary analysis.

### 2.5. Statistical Analysis

All tests were two-sided. Continuous variables were summarized as mean ± SD or median [interquartile range], as appropriate, and categorical variables as number and percentage. Between-group comparisons used Student’s *t* test, Wilcoxon rank-sum test, χ^2^ test, or Fisher exact test as appropriate. Analyses were performed using R version 4.4.3 (R Foundation for Statistical Computing, Vienna, Austria).

### 2.6. Primary Analysis

Associations with prevalent DISH were examined using Firth-penalized logistic regression adjusted for age, sex, and VFA. Immune-cell associations were expressed per 10-percentage-point increase and additionally per one cohort SD to facilitate comparison across subsets with different distributions. Per-SD estimates were obtained by rescaling the same coefficients and confidence limits and did not constitute additional hypothesis tests. Odds ratios (ORs), 95% confidence intervals (CIs), and two-sided *p*-values were reported. The numerical verification used Wald inference based on the information matrix. No immune-cell subset had been designated as a primary exposure before examining its association with DISH. Subsequent analyses of CD16+CD56+ NK cells were therefore considered post hoc and exploratory. Absolute counts were expressed per 100 cells/µL. More extensive adjustment was reserved for sensitivity analyses because only 41 participants had DISH.

### 2.7. Relative Versus Absolute Immune-Cell Representation

Three age-, sex-, and VFA-adjusted Firth models were evaluated: (1) absolute CD16+CD56+ NK-cell count alone, (2) relative CD16+CD56+ NK-cell proportion alone, and (3) absolute count and relative proportion simultaneously. Corresponding maximum-likelihood logistic models were fitted to compare Akaike information criterion (AIC) and area under the receiver operating characteristic curve (AUC) across the base, count, proportion, and count-plus-proportion models, while Firth regression was retained for coefficient estimation and inference.

An exploratory log-ratio analysis contrasted the recorded CD16+CD56+ proportion with the geometric mean of the other three CD16/CD56-defined proportions [16,17]. The balance was calculated as √(3/4) × ln[CD16+CD56+/geometric mean(CD16+CD56−, CD16−CD56+, CD16−CD56−)]. A 0.5-percentage-point pseudocount was added to each component before calculation; pseudocounts of 0.25 and 1.0 were examined in sensitivity analyses. The balance was standardized and analyzed using Firth regression adjusted for age, sex, and VFA. This analysis described a relative contrast among recorded marker-defined components rather than a functional comparison of established NK-cell subsets.

### 2.8. Multiple Testing and Inflammatory-Marker Analyses

Soluble inflammatory markers were evaluated using the same age-, sex-, and VFA-adjusted framework. Benjamini–Hochberg false-discovery-rate (BH-FDR) correction was applied jointly to the eight immune-cell proportions and five soluble inflammatory markers, including CD16+CD56+ NK cells. Adjusted *p*-values were calculated from the unrounded model *p*-values, with a threshold of 0.05. Follow-up analyses of the observed CD16+CD56+ association were exploratory and were not treated as independent confirmation. A sensitivity analysis excluded participants with CRP > 1.0 mg/dL or hs-CRP > 0.3 mg/dL.

### 2.9. Cardiometabolic Adjustment

Potential cardiometabolic confounding was evaluated across glycemic (glucose, insulin), lipid [ln(TG), HDL-C], adiposity, hemodynamic (SBP, DBP), diagnosed metabolic condition, and medication domains. Domain-specific models included age, sex, and the corresponding variables. VFA represented adiposity in the primary model, while BMI and waist circumference were evaluated in separate alternative-adiposity models.

A comprehensive model included age, sex, VFA, fasting glucose, fasting insulin, ln(TG), HDL-C, SBP, and DBP. Additional Firth sensitivity models further incorporated diabetes, dyslipidemia, hypertension, and glucose-lowering, lipid-lowering, and antihypertensive medications. Given the 41 definite-DISH events, estimates from these highly parameterized models were interpreted as sensitivity estimates. Change in the CD16+CD56+ coefficient relative to the age- and sex-adjusted model was summarized descriptively on the log-OR scale as 100 × (βreference − βadjusted)/βreference.

A separate lifestyle sensitivity analysis added smoking history and current alcohol use to the age-, sex-, and VFA-adjusted model. Participants missing either lifestyle variable were excluded from this analysis. The principal model was also refitted in the same complete-case sample to distinguish the effect of additional adjustment from that of sample restriction.

A complementary metabolic-dysregulation score was constructed by equally weighting z-standardized glucose, ln(TG), reverse-coded HDL-C, VFA, and SBP and re-standardizing the composite to mean 0 and SD 1. Clinical metabolic burden was evaluated by comparing participants with none versus at least one diagnosed metabolic condition (diabetes, dyslipidemia, or hypertension), and the interaction between clinical metabolic status and CD16+CD56+ proportion was examined. Exploratory comparisons of model fit and apparent discrimination used AIC, AUC, and likelihood-ratio testing for the comprehensive metabolic model with and without CD16+CD56+ proportion.

### 2.10. Radiographic Phenotype Analyses

CD16+CD56+ proportions were compared across the mutually exclusive none, EDISH, and definite-DISH categories. Age-, sex-, and VFA-adjusted ordinal logistic regression was used to evaluate the ordered none–EDISH–DISH radiographic phenotype. The proportional-odds assumption was assessed by comparing regression coefficients across cumulative logits using a global Wald test; the common OR was reported when this assumption was not violated. Separate Firth logistic models compared EDISH with none and DISH with none. Among participants with DISH, Spearman rank correlation was used to evaluate the association between CD16+CD56+ proportion and OBS.

### 2.11. Robustness Analyses and Diagnostic Procedures

Robustness analyses included replacement of VFA with BMI or waist circumference; exclusion of EDISH from the binary comparator group; exclusion of participants with elevated inflammatory activity; 1000 nonparametric bootstrap repetitions; sequential exclusion of observations with the highest-influence statistics; and exploratory interaction analyses by age and sex. Bootstrap analyses resampled participants with replacement and refitted the primary model in each replicate.

Additional post hoc checks examined the participant-level sum of the four recorded CD16/CD56 proportions. Total lymphocyte count was calculated as white blood cell count (cells/μL) multiplied by the corresponding lymphocyte percentage divided by 100. This count was added to the age-, sex-, and VFA-adjusted Firth model using the same complete-case sample. Variance inflation factors were examined for multicollinearity. Calibration was assessed using the Hosmer–Lemeshow statistic from the corresponding maximum-likelihood logistic model. Missing values were not imputed, and analysis-specific complete cases were used.

## 3. Results

### 3.1. Study Population

All 536 participants in the June 2021 Iwaki examination formed the source sample. Six participants were excluded because of ankylosing spondylitis or postoperative spinal conditions, and four were excluded because their VFA values were recorded as inapplicable, leaving 526 participants for the principal analysis. These comprised 454 participants with neither DISH nor EDISH, 31 with EDISH, and 41 with definite DISH. The binary analysis compared the 41 participants with DISH with 485 participants without definite DISH (Supplementary Figure S1). Smoking and alcohol data were available for 525 participants, including all 41 DISH cases.

Mean age was 62.08 ± 10.65 years. Participants with DISH were not older than those without definite DISH (60.49 ± 11.00 versus 62.21 ± 10.62 years; *p* = 0.469). Women accounted for 30 of 41 DISH cases (73.2%) and 288 of 485 participants without definite DISH (59.4%). Participants with DISH had higher BMI, waist circumference, VFA, SBP, fasting glucose, TG, TyG, and HOMA-IR (Table 1). Median VFA was 84.0 [72.7–103.4] versus 66.8 [52.3–87.3] cm^2^ (*p* = 1.09 × 10^−4^), median SBP was 139.0 [130.5–147.0] versus 123.0 [111.0–136.0] mmHg (*p* = 5.59 × 10^−8^), and hypertension was present in 53.7% versus 20.6% (*p* = 9.64 × 10^−6^), respectively. Inter-reader agreement for DISH classification was excellent (521/526 concordant; κ = 0.932, 95% CI 0.872–0.991), as was OBS reliability (ICC = 0.997, 95% CI 0.996–0.998) (Supplementary Table S1).

### 3.2. Associations of Immune-Cell Subsets and Inflammatory Markers with DISH

**Figure 1:**
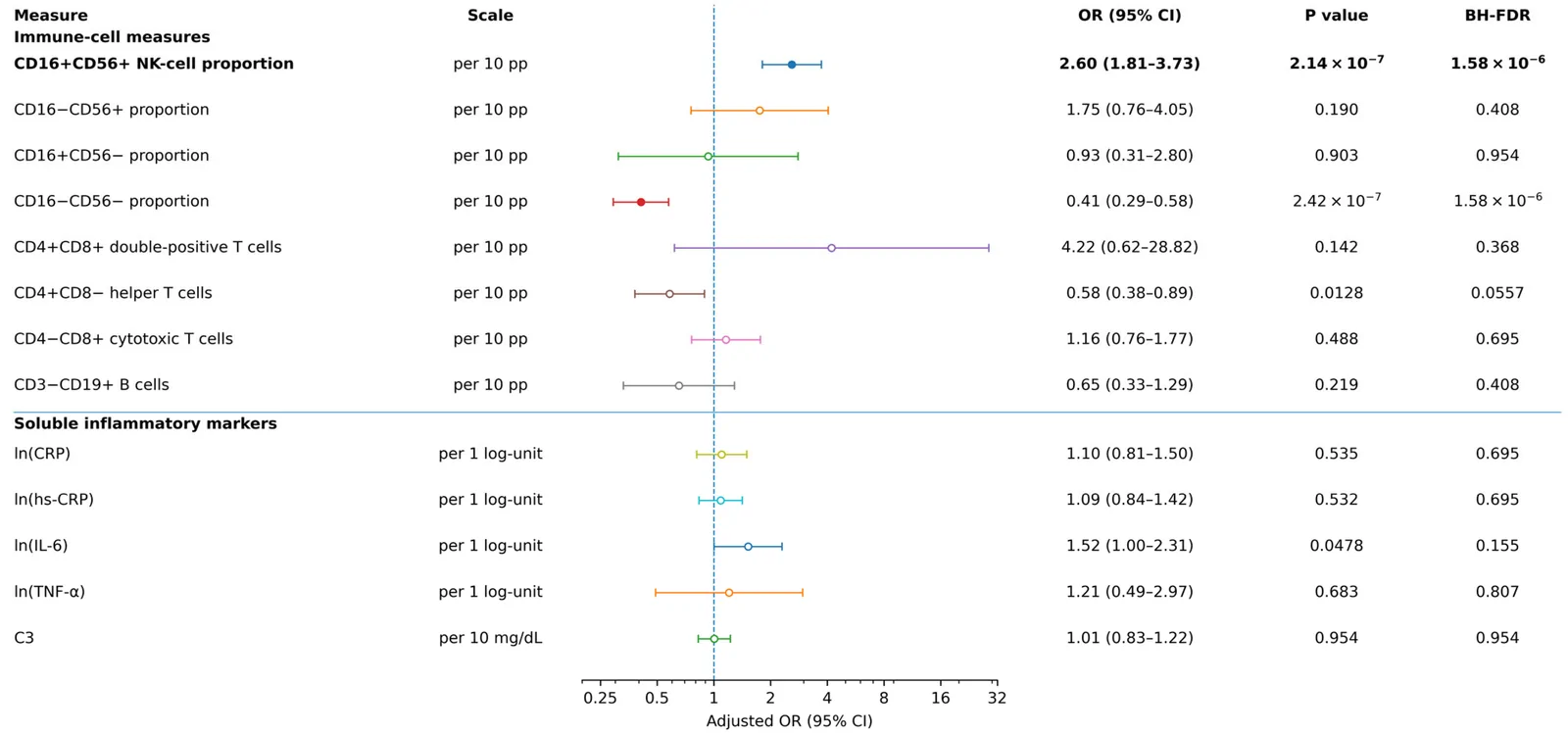
Associations of circulating immune-cell subsets and soluble inflammatory markers with DISH. Points and horizontal intervals represent ORs and 95% CIs from Firth-penalized logistic regression adjusted for age, sex, and VFA. Immune-cell proportions were modeled per 10-percentage-point increase. CRP, hs-CRP, IL-6, and TNF-α were natural-log transformed and modeled per 1-log-unit increase; C3 was modeled per 10 mg/dL increase. BH-FDR correction was applied jointly to all 13 markers, including CD16+CD56+ NK cells. Filled and open markers indicate BH-FDR values below and at or above 0.05, respectively. The horizontal axis is logarithmic, and all confidence intervals are displayed in full. BH-FDR-adjusted *p*-values were calculated from unrounded model *p*-values across all 13 markers, including CD16+CD56+ NK cells.

CD16+CD56+ NK-cell proportion was positively associated with DISH (OR per 10-percentage-point increase, 2.60; 95% CI, 1.81–3.73), whereas CD16−CD56− proportion was inversely associated (OR, 0.41; 95% CI, 0.29–0.58). Both associations remained significant after joint BH-FDR correction across all 13 markers (adjusted *p* = 1.58 × 10^−6^ for each). CD4+CD8− helper T-cell proportion showed a nominal inverse association (OR, 0.58; 95% CI, 0.38–0.89; *p* = 0.0128; adjusted *p* = 0.0557). IL-6 showed a nominal positive association (OR per one-unit increase in ln[IL-6], 1.522; 95% CI, 1.004–2.306; *p* = 0.0478; adjusted *p* = 0.155). Neither association met the FDR threshold of 0.05. No other marker met this threshold (Figure 1; Supplementary Table S2).

### 3.3. Association of CD16+CD56+ Proportion with DISH

**Figure 2:**
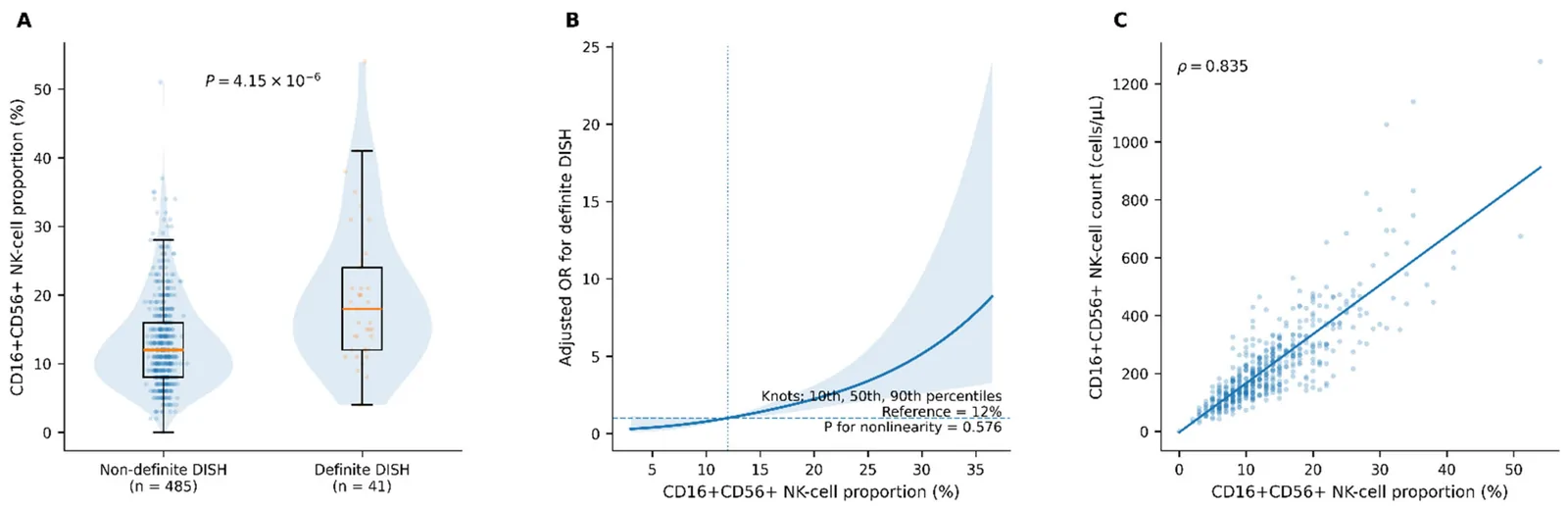
Exploratory characterization of the association between circulating CD16+CD56+ NK cells and DISH. (**A**) CD16+CD56+ NK-cell proportions in participants with and without definite DISH. Violin plots display the distributions, with box plots indicating medians and interquartile ranges and jittered points representing individual observations. (**B**) Restricted cubic spline association estimated using Firth-penalized logistic regression adjusted for age, sex, and VFA. The solid line indicates the adjusted OR and the shaded area the pointwise 95% CI. Knots were placed at 6%, 12%, and 23%, with 12% as the reference. The displayed *p*-value tests departure from linearity. (**C**) Relationship between CD16+CD56+ NK-cell proportion and absolute count, with a fitted linear trend and Spearman correlation coefficient. These complementary analyses characterize associations within the same dataset and do not establish cellular expansion or redistribution.

Median CD16+CD56+ NK-cell proportion was 18% [12–24%] in participants with DISH and 12% [8–16%] in those without definite DISH (*p* = 4.15 × 10^−6^) (Figure 2A). In the age-, sex-, and VFA-adjusted Firth model, each 10-percentage-point increase was associated with higher odds of DISH (OR 2.60, 95% CI 1.81–3.73; *p* = 2.14 × 10^−7^) (Table 2).

A graded association was observed across quartiles. Compared with the lowest quartile, the highest quartile had an OR of 8.39 (95% CI 2.62–26.86; *p* = 3.37 × 10^−4^), and the OR per increasing quartile was 1.92 (95% CI 1.40–2.64; *p* = 5.80 × 10^−5^). Restricted cubic spline analysis showed no evidence of nonlinearity (*p* = 0.576) (Figure 2B; Table 2).

Median absolute CD16+CD56+ count was 276 [165–423] versus 182 [129–273] cells/µL in participants with and without DISH, respectively (*p* = 5.26 × 10^−4^). When modeled separately, each 100-cells/µL increase was associated with DISH (OR 1.41, 95% CI 1.20–1.66; *p* = 2.87 × 10^−5^). Absolute count and relative proportion were strongly correlated (Spearman ρ = 0.835). In the mutually adjusted model, absolute count was not associated with DISH (OR 0.97, 95% CI 0.72–1.30; *p* = 0.816), whereas relative proportion remained associated (OR 2.77 per 10 percentage points, 95% CI 1.47–5.23; *p* = 0.00167) (Table 2; Figure 2C). AIC/AUC values were 283.8/0.693 for the base model, 268.5/0.730 with absolute count, 258.3/0.767 with relative proportion, and 260.2/0.768 with both measures (Table 2).

The standardized compositional log-ratio balance was associated with DISH (OR 1.94 per SD, 95% CI 1.38–2.74; *p* = 1.38 × 10^−4^), with similar estimates using alternative pseudo-counts of 0.25 and 1.0 percentage point (Supplementary Table S3).

### 3.4. Association Across Cardiometabolic Characteristics

In exploratory cardiometabolic sensitivity analyses, the CD16+CD56+ NK-cell estimates remained positive across the alternative adjustment sets (Table 3; Supplementary Figure S2). ORs were 2.46 (95% CI 1.72–3.52; *p* = 7.89 × 10^−7^) after glycemic adjustment, 2.42 (95% CI 1.68–3.49; *p* = 2.01 × 10^−6^) after lipid adjustment, and 2.02 (95% CI 1.40–2.91; *p* = 1.78 × 10^−4^) after hemodynamic adjustment. Alternative BMI and waist-circumference models yielded ORs of 2.44 and 2.47, respectively.

In the comprehensive measured-metabolic model, the OR remained 2.00 (95% CI 1.36–2.94; *p* = 3.91 × 10^−4^). Additional adjustment for diabetes, dyslipidemia, and hypertension yielded an OR of 1.87 (95% CI 1.27–2.75; *p* = 0.00160), and further adjustment for related medications yielded 1.86 (95% CI 1.26–2.75; *p* = 0.00173). Adjustment for the metabolic-dysregulation score yielded an OR of 2.36 (95% CI 1.65–3.36) (Table 3; Supplementary Figure S2).

Among participants without diagnosed diabetes, dyslipidemia, or hypertension, the OR was 3.14 (95% CI 1.71–5.79), compared with 1.85 (95% CI 1.16–2.96) among those with at least one condition; the interaction was not significant (*p* = 0.126). Adding CD16+CD56+ proportion to the comprehensive model reduced AIC from 265.2 to 253.5 and increased AUC from 0.818 to 0.836 (likelihood-ratio *p* = 2.11 × 10^−4^) (Table 3). Among metabolic correlates, SBP showed the strongest association with CD16+CD56+ proportion (+2.14 percentage points per 1 SD; BH-FDR = 2.51 × 10^−9^); DBP, TyG, and ln(TG) were also positively associated after BH-FDR correction, whereas glucose, HOMA-IR, HDL-C, VFA, and fasting insulin were not (Supplementary Table S4).

### 3.5. CD16+CD56+ Composition Across Radiographic Phenotypes

**Figure 3:**
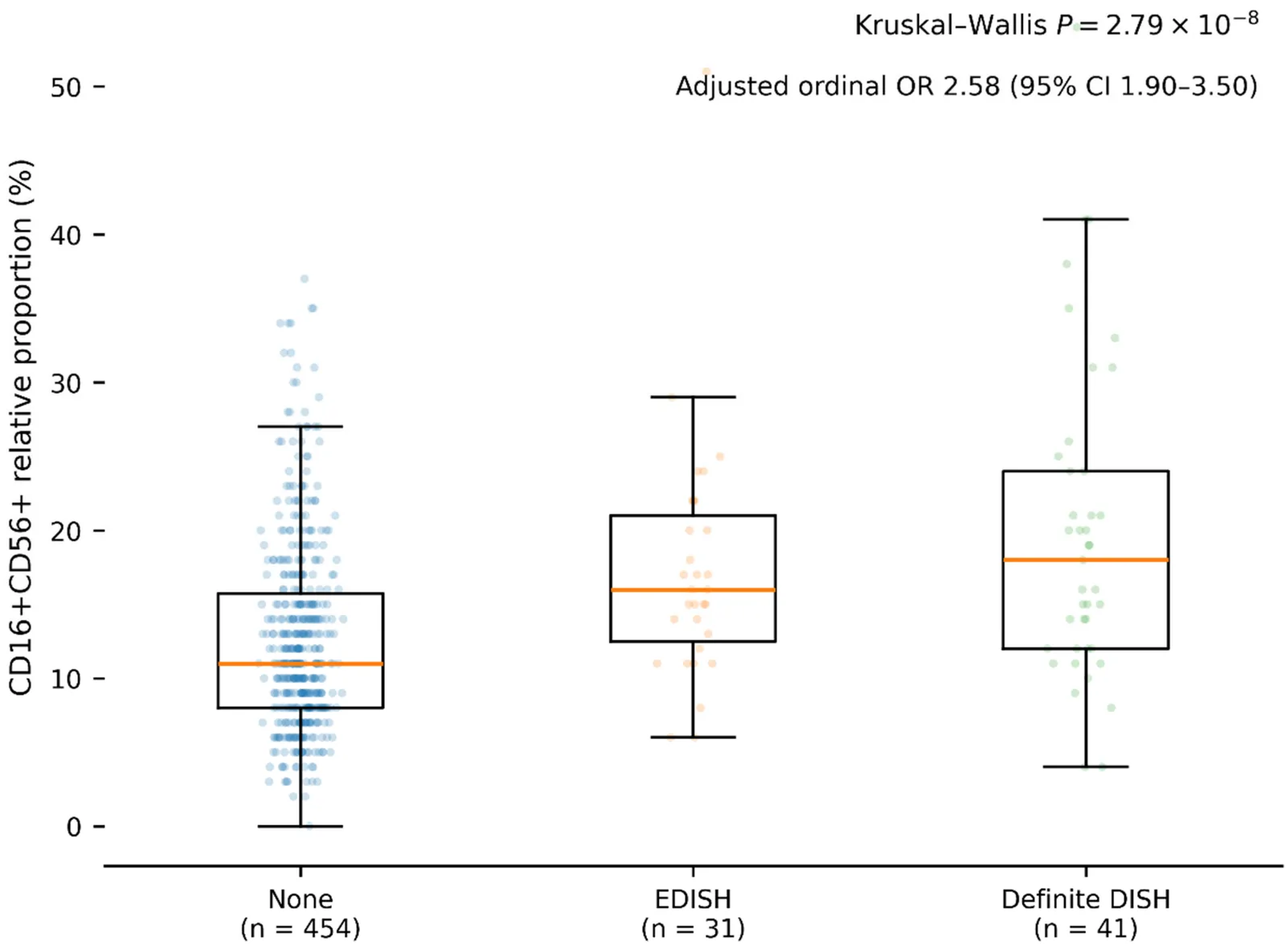
CD16+CD56+ NK-cell proportions across cross-sectional radiographic categories. Box plots show medians and interquartile ranges, with jittered points representing individual observations. “None” denotes participants with neither DISH nor EDISH. Overall differences were evaluated using the Kruskal–Wallis test. The displayed common OR and 95% CI were obtained from ordinal logistic regression adjusted for age, sex, and VFA. The categories comprise different participants assessed at a single examination and do not represent observed longitudinal progression.

Median CD16+CD56+ NK-cell proportions were 11% [8–15.75%], 16% [12.5–21%], and 18% [12–24%] in participants with neither DISH nor EDISH, EDISH, and definite DISH, respectively (*p* = 2.79 × 10^−8^) (Figure 3; Supplementary Table S5). Each 10-percentage-point increase was associated with EDISH versus none (OR 2.16, 95% CI 1.40–3.35) and definite DISH versus none (OR 2.86, 95% CI 1.96–4.17). The proportional-odds assumption was not violated (global *p* = 0.250), and the corresponding common OR for a higher radiographic category was 2.58 (95% CI 1.90–3.50; *p* = 1.04 × 10^−9^). Among participants with definite DISH, neither relative proportion (ρ = 0.196, *p* = 0.220) nor absolute count (ρ = 0.256, *p* = 0.106) was associated with OBS (Supplementary Table S5).

### 3.6. Robustness and Model Diagnostics

Among 525 participants with complete lifestyle data, further adjustment for smoking history and current alcohol use yielded an OR of 2.61 per 10-percentage-point increase in CD16+CD56+ NK-cell proportion (95% CI, 1.82–3.73; *p* = 1.64 × 10^−7^). The corresponding age-, sex-, and VFA-adjusted estimate in the same sample was 2.63 (95% CI, 1.83–3.78).

The four recorded CD16/CD56 proportions summed to 97–100%, with a median of 98% [98–99%], rather than uniformly totaling 100%. Additional adjustment for total lymphocyte count yielded a similar association with DISH (OR per 10-percentage-point increase, 2.59; 95% CI, 1.81–3.71; *p* = 2.24 × 10^−7^; Supplementary Table S6).

The primary association remained stable across alternative-adiposity adjustment, exclusion of EDISH, inflammatory-marker exclusion, bootstrap resampling, and influence analyses. Across 1000 bootstrap samples, the median OR was 2.65 with a 95% percentile confidence interval of 1.87–3.95, and sequential exclusion of the 10 highest-influence observations yielded ORs of 2.47–2.84 (Supplementary Figure S3; Supplementary Table S4). The conventional primary model had AUC 0.767 and Hosmer–Lemeshow *p* = 0.585. The maximum VIF was 1.01 in the primary model and 3.31 and 3.29 for absolute count and relative proportion, respectively, in the mutually adjusted model. Age and sex interactions were not significant (Supplementary Table S4).

## 4. Discussion

This exploratory study identified differences in circulating immune-cell proportions associated with prevalent DISH. The positive CD16+CD56+ NK-cell association and inverse CD16−CD56− association remained significant after adjustment for age, sex, and VFA and correction across the full marker panel. The helper T-cell and IL-6 findings were nominal and require cautious interpretation. Follow-up analyses suggested that relative cellular representation carried information beyond absolute CD16+CD56+ count, but these analyses were conducted in the same dataset and do not provide independent validation.

Previous studies have linked DISH to metabolic syndrome, cardiovascular morbidity, insulin resistance, and alterations in inflammatory or bone-regulatory mediators [4,5,6,7,10,11,12,13]. Our findings add a circulating cellular dimension to this systemic phenotype. None of the soluble inflammatory markers met the multiple-testing threshold, and exclusion of participants with elevated CRP or hs-CRP yielded a similar NK-cell estimate. Nevertheless, this does not establish that the cellular association is independent of all inflammatory processes, specific to DISH, or representative of immune activity within spinal ligaments and entheses.

Relative proportions and absolute counts are mathematically related, and their mutually adjusted associations require careful interpretation. The retained association with CD16+CD56+ proportion describes its conditional statistical relationship with DISH, not evidence of preferential NK-cell production, survival, or recruitment. The log-ratio analysis similarly describes a relative contrast within the recorded CD16/CD56 profile [16,17]. Changes in other circulating populations may contribute to both findings. Although the association persisted after adjustment for total lymphocyte count, the approximately constant sum of the recorded proportions limits independent interpretation of individual components. The inverse CD16−CD56− association should therefore not be interpreted as a protective cellular mechanism, particularly because the available metadata did not establish the lineage composition of that component. The nominal helper T-cell association likewise does not establish an independent protective effect.

NK cells respond to metabolic cues, and experimental studies have linked their activity to adipose inflammation, insulin resistance, and obesity-associated functional changes [9,21,22]. Their effects on bone are also context-dependent: NK cells can promote osteoclast formation in inflammatory arthritis, whereas activated NK cells can kill mature osteoclasts under other experimental conditions [14,15]. These observations support biological plausibility for an immune–metabolic–skeletal connection, but neither direction of effect can be inferred from circulating cell proportions in DISH. NK-cell phenotype and function vary with maturation and tissue distribution [23,24,25], and this study did not measure activation, cytotoxicity, cytokine production, or localization at ossifying sites. A higher circulating CD16+CD56+ proportion should therefore not be equated with enhanced NK-cell activity or direct participation in pathological bone formation.

Blood pressure warrants particular attention because hemodynamic adjustment produced the largest reduction among the domain-specific estimates, and SBP showed the strongest association with CD16+CD56+ proportion among the metabolic correlates examined. Population-based research has also linked circulating NK-cell measures to blood pressure [26]. Blood pressure may therefore act as a confounder or mark shared cardiometabolic processes associated with both immune-cell composition and DISH. These explanations cannot be distinguished in a cross-sectional analysis. Changes in logistic coefficients also reflect model specification and noncollapsibility and cannot quantify mediation or the proportion of an association explained. The similar estimate after adjustment for smoking and alcohol use provides additional evidence of robustness to these measured lifestyle factors, but does not eliminate residual confounding. Given the small number of DISH cases, the age-, sex-, and VFA-adjusted analysis remains the principal basis for inference; the more extensively adjusted analyses remain exploratory sensitivity assessments.

CD16+CD56+ NK-cell proportions differed across radiographic categories, but these comparisons involved different individuals assessed at one examination. Although EDISH criteria and longitudinal imaging research provide a framework for studying early radiographic changes [19,27], the present findings do not demonstrate progression from EDISH to DISH. The absence of a detectable association with OBS among established cases also does not support interpretation as a marker of ossification burden, although precision was limited. More broadly, this study did not evaluate prospective disease development, progression, treatment response, or diagnostic performance in an independent population. The findings therefore identify an exploratory circulating correlate of prevalent DISH, not an established diagnostic or prognostic biomarker, disease-activity measure, or therapeutic target. Lower AIC and higher apparent AUC represent within-sample model comparisons only.

Strengths include the community-based setting, integrated radiographic and clinical information, high inter-reader agreement, and complementary analyses of relative and absolute cellular measures. Several limitations remain. The cross-sectional design precludes temporal and causal inference. Only 41 participants had DISH, limiting precision and the reliability of highly adjusted and subgroup estimates despite Firth penalization. The marker screen and subsequent NK-cell analyses were exploratory and require independent replication. Women constituted 73.2% of DISH cases, and cases were not older than participants without definite DISH, differing from the older male predominance commonly described in DISH [1]. This pattern may reflect volunteer participation, the demographic composition of the examination sample, or sampling variability; its cause cannot be established here. Age and sex adjustment does not restore population representativeness. Laboratory data were obtained from a single examination, and incomplete assay and phenotyping metadata limit assessment of measurement comparability and cellular specificity. Smoking and alcohol use were addressed in sensitivity analysis, but residual confounding remains possible from exposure intensity, physical activity, recent infection, chronic viral exposure, and immune-modifying medications. Cytomegalovirus can leave persistent imprints on the NK-cell repertoire [28] and was not evaluated. CRP-based exclusions do not resolve this limitation. Finally, database availability, complete-case selection, and recruitment from one Japanese community may limit generalizability.

## 5. Conclusions

In this community-based cross-sectional analysis, DISH was associated with higher CD16+CD56+ NK-cell and lower CD16−CD56− proportions after adjustment for age, sex, and VFA and correction for multiple testing. The helper T-cell and IL-6 associations were nominal. These exploratory findings suggest that differences in circulating immune-cell composition accompany the cardiometabolic phenotype of DISH, but do not establish disease specificity, a causal mechanism, or clinical biomarker utility. Independent studies with detailed immunophenotyping and longitudinal assessment are needed to clarify their biological significance.

## Figures and Tables

**Table 1:** Characteristics of participants according to definite-DISH status.

Characteristic	Overall (*n* = 526)	Non-DISH (*n* = 485)	DISH (*n* = 41)	*p* Value
Demographic and lifestyle characteristics				
Age, years	62.08 ± 10.65	62.21 ± 10.62	60.49 ± 11.00	0.469
Female sex, *n* (%)	318 (60.5)	288 (59.4)	30 (73.2)	0.097
Smoking history, *n* (%)	253 (48.1)	234 (48.2)	19 (46.3)	0.872
Current alcohol use, *n* (%)	269 (51.1)	246 (50.7)	23 (56.1)	0.521
Anthropometric and hemodynamic characteristics				
BMI, kg/m^2^	22.6 [20.5–24.9]	22.4 [20.3–24.7]	24.6 [22.9–26.4]	7.36 × 10^−6^
Waist circumference, cm	80.3 [74.8–87.2]	79.8 [74.4–86.4]	87.5 [78.8–91.6]	1.47 × 10^−4^
VFA, cm^2^	68.8 [52.7–90.6]	66.8 [52.3–87.3]	84.0 [72.7–103.4]	1.09 × 10^−4^
SBP, mmHg	124.2 [112.5–137.5]	123.0 [111.0–136.0]	139.0 [130.5–147.0]	5.59 × 10^−8^
DBP, mmHg	79.5 [72.0–87.0]	79.0 [71.5–87.0]	85.0 [79.5–87.0]	0.0042
Metabolic characteristics				
Fasting glucose, mg/dL	96 [90–102]	95 [90–101]	104 [96–117]	1.32 × 10^−5^
Fasting insulin, μU/mL	4.4 [3.1–6.1]	4.2 [3.1–6.0]	5.5 [3.7–6.7]	0.023
TG, mg/dL	80 [58–123]	78 [57–122]	104 [85–136]	0.0011
HDL-C, mg/dL	67 [55–79.8]	67 [56–80]	65 [54–73]	0.446
TyG index	8.28 [7.89–8.72]	8.24 [7.88–8.70]	8.57 [8.38–9.04]	1.65 × 10^−4^
HOMA-IR	1.04 [0.73–1.52]	1.03 [0.72–1.46]	1.52 [0.90–1.91]	0.0026
Diabetes, *n* (%)	33 (6.3)	31 (6.4)	2 (4.9)	1.000
Dyslipidemia, *n* (%)	92 (17.5)	82 (16.9)	10 (24.4)	0.282
Hypertension, *n* (%)	122 (23.2)	100 (20.6)	22 (53.7)	9.64 × 10^−6^
Selected immune-cell measures				
CD16+CD56+ proportion, %	12 [8–17]	12 [8–16]	18 [12–24]	4.15 × 10^−6^
CD16+CD56+ count, cells/μL	185 [132–280]	182 [129–273]	276 [165–423]	5.26 × 10^−4^
Total lymphocyte count, cells/μL	1600 [1326–1976]	1600 [1326–1976]	1645 [1175–2014]	0.768

Values are mean ± SD, median [interquartile range], or *n* (%), as appropriate. *p*-values were obtained using Student’s *t*-test or the Wilcoxon rank-sum test for continuous variables and the χ^2^ test or Fisher exact test for categorical variables, as appropriate. Smoking history included current and former smoking. EDISH participants were included in the non-definite-DISH group for the primary binary comparison. BMI, body mass index; DBP, diastolic blood pressure; DISH, diffuse idiopathic skeletal hyperostosis; EDISH, early-phase DISH; HDL-C, high-density lipoprotein cholesterol; HOMA-IR, Homeostatic Model Assessment for Insulin Resistance; SBP, systolic blood pressure; TG, triglycerides; TyG, triglyceride–glucose index; VFA, visceral fat area.

**Table 2:** Associations of relative and absolute CD16+CD56+ NK-cell measures with definite DISH.

Analysis	Exposure Specification	OR (95% CI)	*p* Value	AIC	AUC
Relative-proportion analysis	Per 10-percentage-point increase	2.60 (1.81–3.73)	2.14 × 10^−7^	258.3	0.767
Quartile analysis	Q1	Reference	—	—	—
	Q2 vs. Q1	2.94 (0.84–10.24)	0.091	—	—
	Q3 vs. Q1	3.13 (0.88–11.16)	0.078	—	—
	Q4 vs. Q1	8.39 (2.62–26.86)	3.37 × 10^−4^	—	—
	Per increasing quartile	1.92 (1.40–2.64)	5.80 × 10^−5^	—	—
Restricted cubic spline	Departure from linearity	—	0.576	—	—
Absolute-count analysis	Per 100 cells/μL	1.41 (1.20–1.66)	2.87 × 10^−5^	268.5	0.730
Mutually adjusted model	Absolute count, per 100 cells/μL	0.97 (0.72–1.30)	0.816	260.2	0.768
	Relative proportion, per 10 percentage points	2.77 (1.47–5.23)	0.00167	—	—
Compositional log-ratio analysis	Balance, per 1 SD	1.94 (1.38–2.74)	1.38 × 10^−4^	—	—
Exploratory model fit and discrimination					
Age + sex + VFA only	Not applicable	—	—	283.8	0.693
+ Absolute CD16+CD56+ count	Per 100 cells/μL	—	—	268.5	0.730
+ CD16+CD56+ proportion	Per 10-percentage-point increase	—	—	258.3	0.767
+ Absolute count and proportion	Both measures	—	—	260.2	0.768

Odds ratios were estimated using Firth-penalized logistic regression adjusted for age, sex, and VFA. AIC and AUC were obtained from the corresponding conventional maximum-likelihood logistic regression models because Firth regression was used for coefficient estimation and inference. Relative proportion was modeled per 10-percentage-point increase and absolute count per 100 cells/μL. Quartiles were defined from the cohort distribution of CD16+CD56+ proportion. The compositional balance contrasted CD16+CD56+ with the geometric mean of the remaining three CD16/CD56-defined components and was standardized before modeling. The restricted cubic spline used three knots at the 10th, 50th, and 90th percentiles of the CD16+CD56+ NK-cell proportion distribution (6%, 12%, and 23%, respectively), with 12% as the reference; departure from linearity was assessed using a Wald test of the nonlinear spline term. AUC, area under the receiver operating characteristic curve; CI, confidence interval; OR, odds ratio.

**Table 3:** Exploratory cardiometabolic sensitivity analyses of the association between CD16+CD56+ NK-cell proportion and DISH.

Model	Adjustment Variables	OR per 10-Percentage-Point Increase (95% CI)	*p* Value	Log-OR Attenuation, % *
Demographic model	Age, sex	2.53 (1.77–3.62)	3.53 × 10^−7^	Reference
Primary model	Age, sex, VFA	2.60 (1.81–3.73)	2.14 × 10^−7^	−2.8
Glycemic domain	Age, sex, fasting glucose, fasting insulin	2.46 (1.72–3.52)	7.9 × 10^−7^	3.0
Lipid domain	Age, sex, ln(TG), HDL-C	2.42 (1.68–3.49)	2.01 × 10^−6^	4.6
Hemodynamic domain	Age, sex, SBP, DBP	2.02 (1.40–2.91)	1.78 × 10^−4^	24.5
Alternative adiposity	Age, sex, BMI	2.44 (1.70–3.49)	1.10 × 10^−6^	4.0
Alternative adiposity	Age, sex, waist circumference	2.47 (1.73–3.53)	6.34 × 10^−7^	2.4
Comprehensive measured-metabolic model	Age, sex, VFA, glucose, insulin, ln(TG), HDL-C, SBP, DBP	2.00 (1.36–2.94)	3.91 × 10^−4^	25.4
+ Clinical metabolic diagnoses	Above + diabetes, dyslipidemia, hypertension	1.87 (1.27–2.75)	0.00160	32.6
+ Metabolic treatment	Above + glucose-lowering, lipid-lowering, antihypertensive medication	1.86 (1.26–2.75)	0.00173	33.2
Metabolic-dysregulation score	Age, sex + composite metabolic score	2.36 (1.65–3.36)	2.15 × 10^−6^	—
Panel B. Clinical metabolic burden				
Clinical metabolic status	*n*	DISH, *n*	OR (95% CI)	*p* value
No diagnosed diabetes, dyslipidemia, or hypertension	353	13	3.14 (1.71–5.79)	2.41 × 10^−4^
≥1 diagnosed metabolic condition	172	28	1.85 (1.16–2.96)	0.0098
Interaction	—	—	—	0.126
Panel C. Incremental model information				
Model	AIC	AUC	Likelihood-ratio *p*-value	
Comprehensive metabolic model	265.2	0.818	Reference	
Comprehensive metabolic model + CD16+CD56+ proportion	253.5	0.836	2.11 × 10^−4^	

Firth-penalized logistic regression was used for OR estimation. * Attenuation was calculated relative to the age- and sex-adjusted model on the log-OR scale as 100 × (β_reference_ − β_adjusted_)/β_reference_. Negative attenuation indicates a slightly larger coefficient after adjustment. The metabolic-dysregulation score was constructed by equally weighting z-standardized fasting glucose, ln(TG), reverse-coded HDL-C, VFA, and SBP and subsequently re-standardizing the composite to a mean of 0 and an SD of 1. Higher scores indicate greater metabolic dysregulation. One participant with an indeterminate clinical metabolic-disease entry was excluded from the clinical-burden stratification. AIC and AUC for incremental modeling were obtained from conventional logistic regression.

## Data Availability

The datasets analyzed during the current study are not publicly available because they contain participant-level information subject to privacy protections and restrictions under the ethical approval and informed consent governing the Iwaki Health Promotion Project. Data may be available from the corresponding author upon reasonable request and with appropriate permission from the relevant ethics committee and the Iwaki Health Promotion Project.

## Supplementary Materials

The following supporting information can be downloaded at: https://www.mdpi.com/article/10.3390/jcm15218458/s1. Table S1: Inter-reader reliability of radiographic assessments; Table S2: Immune-cell and inflammatory-marker associations with DISH and inflammatory-exclusion analyses; Table S3: Exploratory log-ratio analyses of CD16/CD56-defined cell proportions; Table S4: Metabolic correlates, robustness analyses, and model diagnostics; Table S5: Association of CD16+CD56+ NK-cell composition with radiographic DISH phenotypes and ossification burden; Table S6: Checks of CD16/CD56 component proportions and sensitivity analysis adjusting for total lymphocyte count; Figure S1: Participant selection; Figure S2: Exploratory cardiometabolic sensitivity analyses; Figure S3: Internal robustness analyses.
