# Supplementary material for: Circulating Immune-Cell Profiles and Diffuse Idiopathic Skeletal Hyperostosis: A Community-Based Cross-Sectional Study
Source: J Clin Med. 2026 Oct 30;15(21):8458. doi: 10.3390/jcm15218458 (PMC13764354; doi:10.3390/jcm15218458)
Supplement: Supplementary file 1 [file jcm-15-08458-s001.zip › jcm-4641167-supplementary.pdf]

Supplementary Table S1. Inter-reader reliability of radiographic assessments.

Assessment	Reliability measure	Estimate	95% CI / additional measure
DISH classification	Exact agreement	521/526 (99.0%)	—
DISH classification	Cohen's κ	0.932	0.872–0.991
DISH classification	Positive agreement	0.937	—
DISH classification	Negative agreement	0.995	—
DISH classification	McNemar test	—	P=1.00
Ossification burden score	ICC(A,1)	0.997	0.996–0.998
Ossification burden score	Bland–Altman mean bias	−0.121	—
Ossification burden score	95% limits of agreement	—	−1.31 to 1.07

Values are presented as agreement counts (percentages), reliability coefficients with 95% confidence intervals (CIs), or P values, as appropriate. Agreement for DISH classification was assessed using Cohen's κ, with positive and negative agreement additionally reported and marginal disagreement evaluated using McNemar's test. Reliability of the ossification burden score was assessed using a two-way random-effects, absolute-agreement, single-measure intraclass correlation coefficient [ICC(A,1)]. Bland–Altman analysis was used to quantify the mean inter-reader difference and 95% limits of agreement. CI, confidence interval; DISH, diffuse idiopathic skeletal hyperostosis; ICC, intraclass correlation coefficient.

Supplementary Table S2. Immune-cell and inflammatory-marker associations with DISH and inflammatory-exclusion analyses.

Marker or analysis	Exposure scale / analysis population	Adjusted OR (95% CI)	P value	BH-FDR
Immune-cell measures				
CD16+CD56+ NK-cell proportion	Per 10-percentage-point increase	2.01 (1.55–2.62)	2.14×10^{-7}	1.58×10^{-6}
CD16−CD56+ proportion	Per 10-percentage-point increase	1.21 (0.91–1.62)	0.190	0.408
CD16+CD56− proportion	Per 10-percentage-point increase	0.98 (0.72–1.34)	0.903	0.954
CD16−CD56− proportion	Per 10-percentage-point increase	0.49 (0.37–0.64)	2.42×10^{-7}	1.58×10^{-6}
CD4+CD8+ double-positive T cells	Per 10-percentage-point increase	1.21 (0.94–1.55)	0.142	0.368
CD4+CD8− helper T cells	Per 10-percentage-point increase	0.66 (0.47–0.91)	0.0128	0.0557
CD4−CD8+ cytotoxic T cells	Per 10-percentage-point increase	1.12 (0.82–1.53)	0.488	0.695
CD3−CD19+ B cells	Per 10-percentage-point increase	0.80 (0.56–1.14)	0.219	0.408
Soluble inflammatory markers				
ln(CRP)	Per 1-log-unit increase	1.10 (0.81–1.50)	0.535	0.695
ln(hs-CRP)	Per 1-log-unit increase	1.09 (0.84–1.42)	0.532	0.695
ln(IL-6)	Per 1-log-unit increase	1.52 (1.00–2.31)	0.0478	0.155
ln(TNF-α)	Per 1-log-unit increase	1.21 (0.49–2.97)	0.683	0.807
C3	Per 10-mg/dL increase	1.01 (0.83–1.22)	0.954	0.954
Inflammatory-activity sensitivity analysis				
Excluding participants with CRP >1.0 mg/dL or hs-CRP >0.3 mg/dL	CD16+CD56+ NK-cell proportion, per 10-percentage-point increase	2.55 (1.76–3.70)	8.75×10^{-7}	—

Estimates were obtained using Firth penalized logistic regression adjusted for age, sex, and VFA. Immune-cell proportions are reported per 10-percentage-point increase and per one cohort SD; both scales describe the same fitted association. CRP, hs-CRP, IL-6, and TNF-α were natural-log transformed, and C3 was expressed per 10-mg/dL increase. BH-FDR correction was applied jointly to all eight immune-cell proportions and five inflammatory markers, including CD16+CD56+ NK cells, using unrounded model P values. The inflammatory-exclusion analysis was exploratory and was not included as an additional test in this family.

Supplementary Table S3. Exploratory log-ratio analyses of CD16/CD56-defined cell proportions.

Analysis	Zero-replacement/pseudo-count specification	OR per 1 SD (95% CI)	P value
Balance with a 0.50-percentage-point pseudo-count	+0.50 percentage point to each component	1.94 (1.38–2.74)	1.38×10^{-4}
Sensitivity analysis	+0.25 percentage point to each component	1.91 (1.35–2.70)	2.32×10^{-4}
Sensitivity analysis	+1.00 percentage point to each component	1.98 (1.42–2.78)	6.86×10^{-5}

The balance was defined as √(3/4)×ln[CD16+CD56+ / geometric mean(CD16+CD56−, CD16−CD56+, CD16−CD56−)] and standardized before analysis. Models were fitted using Firth penalized logistic regression adjusted for age, sex, and VFA.

Supplementary Table S4. Metabolic correlates, robustness analyses, and model diagnostics.

Metabolic characteristic	Change in CD16+CD56+ proportion per 1 SD, percentage points (95% CI)	P value	BH-FDR
SBP	+2.14 (1.48 to 2.80)	2.28×10^{-10}	2.51×10^{-9}
DBP	+1.05 (0.43 to 1.67)	8.28×10^{-4}	0.0046
TyG	+0.98 (0.31 to 1.66)	0.0041	0.015
ln(TG)	+0.82 (0.14 to 1.49)	0.0180	0.049
BMI	+0.69 (0.08 to 1.30)	0.0277	0.061
Waist circumference	+0.54 (−0.05 to 1.12)	0.0719	0.132
Fasting glucose	+0.74 (−0.35 to 1.83)	0.183	0.287
ln(HOMA-IR)	+0.29 (−0.35 to 0.92)	0.376	0.517
HDL-C	+0.20 (−0.45 to 0.84)	0.551	0.673
VFA	+0.09 (−0.46 to 0.65)	0.736	0.810
Fasting insulin	+0.07 (−0.53 to 0.67)	0.819	0.819

Additional robustness analyses

Analysis	Result
Bootstrap resampling, 1,000 repetitions	Median OR 2.65; percentile 95% interval 1.87–3.95
Sequential exclusion of 10 highest-influence observations	OR range 2.47–2.84
Exclusion of EDISH from binary comparator group	OR 2.86 (1.96–4.17); $P=5.39\times10^{-8}$
Primary conventional logistic model	AUC 0.767
Hosmer-Lemeshow calibration test	P=0.585
Maximum VIF, primary model	1.01
VIF for absolute count in mutually adjusted model	3.31
VIF for relative proportion in mutually adjusted model	3.29
Sex × CD16+CD56+ interaction	P=0.322
Age × CD16+CD56+ interaction	P=0.076

Supplementary Table S5. Association of CD16+CD56+ NK-cell composition with radiographic DISH phenotypes and ossification burden.

Panel A. CD16+CD56+ proportion across radiographic phenotypes			
Radiographic phenotype	n	CD16+CD56+ proportion, %	Overall P value
Neither DISH nor EDISH	454	11 [8–15.75]	
EDISH	31	16 [12.5–21]	
DISH	41	18 [12–24]	2.79×10^{-8}

Panel B. Adjusted radiographic phenotype models			
Outcome comparison	Exposure	OR (95% CI)	P value
EDISH vs none	CD16+CD56+ proportion, per 10 percentage points	2.16 (1.40–3.35)	5.40×10^{-4}
DISH vs none	CD16+CD56+ proportion, per 10 percentage points	2.86 (1.96–4.17)	5.39×10^{-8}
Ordered radiographic categories: neither DISH nor EDISH, EDISH, and DISH	CD16+CD56+ proportion, per 10 percentage points	2.58 (1.90–3.50)	1.04×10^{-9}
Proportional-odds assumption	Global test	—	0.250

Panel C. Ossification burden among participants with DISH		
Analysis	Estimate	P value
OBS, median [IQR]	5 [3–9]	—
CD16+CD56+ proportion vs OBS	Spearman ρ=0.196	0.220
Absolute CD16+CD56+ count vs OBS	Spearman ρ=0.256	0.106

Values are median [interquartile range] unless otherwise indicated. Between-group comparison across the three radiographic categories used the Kruskal-Wallis test. Pairwise binary associations were estimated using Firth logistic regression adjusted for age, sex, and VFA. The ordered radiographic phenotype was evaluated using age-, sex-, and VFA-adjusted ordinal logistic regression. OBS analyses were restricted to participants with DISH. EDISH, early-phase diffuse idiopathic skeletal hyperostosis; OBS, ossification burden score.

Supplementary Table S6. Checks of CD16/CD56 component proportions and sensitivity analysis adjusting for total lymphocyte count.

Check or analysis	Result
Participants included	526, including 41 DISH cases
Sum of four CD16/CD56 proportions, mean ± SD	98.19 ± 0.56%
Sum, median [IQR]; range	98 [98 – 99]%; 97 – 100%
Participants with sums of 97%, 98%, 99%, and 100%	39, 347, 139, and 1, respectively
Age-, sex-, and VFA-adjusted association	OR 2.60; 95% CI 1.81 – 3.73; $P=2.14\times10^{-7}$
Additional adjustment for total lymphocyte count	OR 2.59; 95% CI 1.81 – 3.71; $P=2.24\times10^{-7}$

ORs describe a 10-percentage-point increase in CD16+CD56+ proportion. Total lymphocyte count was calculated from the corresponding white blood cell count and lymphocyte percentage and entered per 1000 cells/µL. Both models used the same participants. Confidence intervals and two-sided P values were obtained using Wald inference from Firth penalized logistic regression. These post hoc checks were supplementary to the original 13-marker screening family.

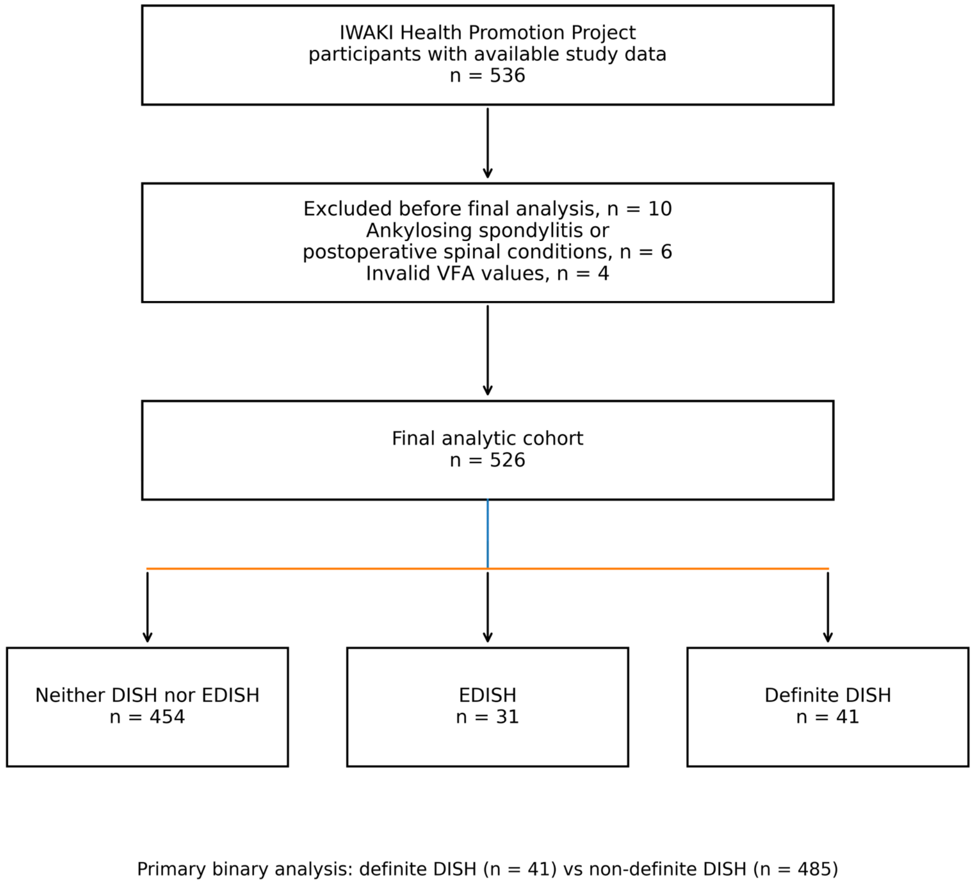

Supplementary Figure S1. Participant selection from the June 2021 Iwaki Health Promotion Project examination. Of 536 participants, six were excluded because of ankylosing spondylitis or postoperative spinal conditions and four because of invalid VFA entries, leaving 526 participants. These comprised 454 participants with neither DISH nor EDISH, 31 with EDISH, and 41 with definite DISH. EDISH was included in the comparator group for the binary analysis.

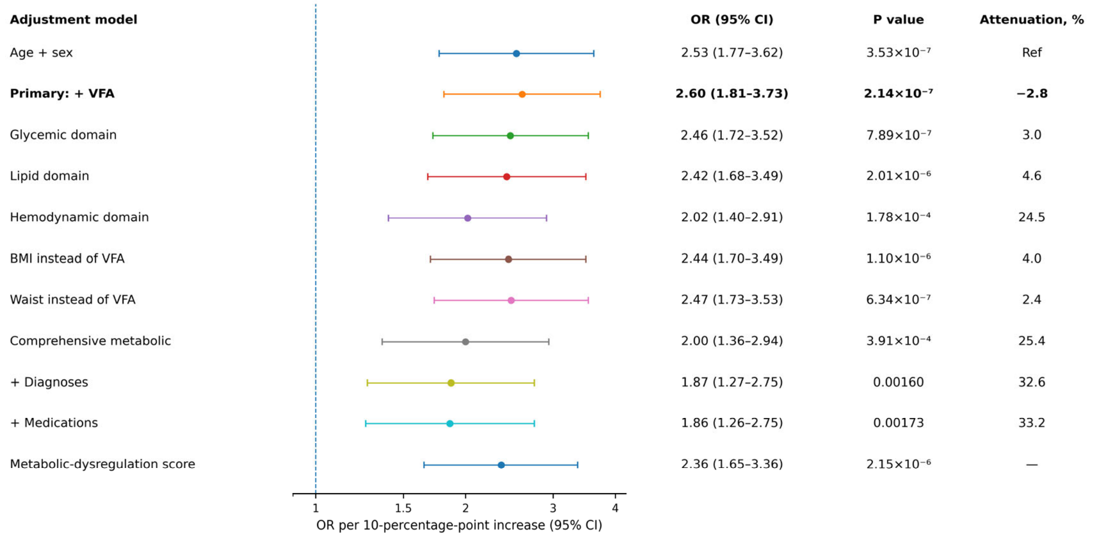

Supplementary Figure S2. **Exploratory cardiometabolic sensitivity analyses of the CD16+CD56+ NK-cell association with DISH.** ORs and 95% CIs were estimated using Firth penalized logistic regression and are expressed per 10-percentage-point increase in CD16+CD56+ NK-cell proportion. The age-, sex-, and VFA-adjusted estimate is highlighted as the principal basis for inference. Other adjustment sets are exploratory sensitivity analyses; their covariates are specified in Table 3. Percentage change in the log-OR was calculated relative to the age- and sex-adjusted estimate as 100 × (βreference − βadjusted)/βreference. This descriptive coefficient change does not quantify mediation or the proportion of the association explained by additional covariates.

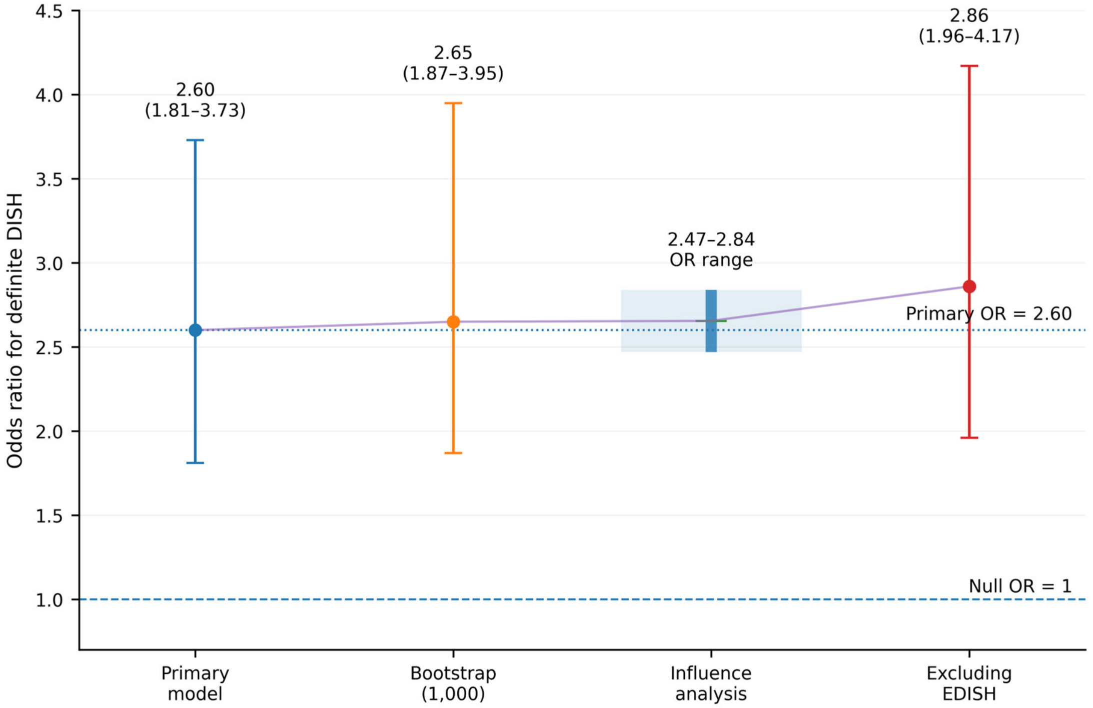

Supplementary Figure S3. **Internal robustness of the CD16+CD56+ NK-cell association with DISH.** The figure displays the age-, sex-, and VFA-adjusted Firth estimate, bootstrap results from 1,000 resamples, the range of estimates after sequential exclusion of the 10 highest-influence observations, and the estimate after exclusion of EDISH from the comparator group. Intervals represent 95% CIs for the fitted analyses and a 95% percentile interval for bootstrap resampling. The influence-analysis segment represents the range of OR estimates, not a confidence interval; no midpoint estimate is plotted. The dashed line denotes OR=1, and the dotted line denotes the principal adjusted estimate of 2.60. These analyses assess internal robustness rather than independent validation.
